# Society Meetings

**Published:** 1911-06

**Authors:** 


### SOCIETY MEETINGS

THE Rochester Academy of Medicine held meetings during May as follows:

*Section of General Medicine.*—Wednesday evening, May 3. Program: Ethics of medical practice, Professor E. G. Frazier, University of Rochester (by invitation).

*Section of Surgery.*—Wednesday evening, May 10. Program: Infection of the Labyrinth, B. A. Richards. Discussion was opened by N. D. McDowell.

*Section of Obstetrics.*—Wednesday evening, May 17. Program: The treatment of urethral gonorrhea in women, E. Wood Ruggles; Chronic gonorrheal urethritis in the male, D. F. MacDonell.

*Section of Hygiene.*—Wednesday evening, May 24. Program: The Iola sanitarium, Montgomery Leary.

THE Buffalo Academy of Medicine held meetings during May as follows:

Special memorial meeting Tuesday evening, May 2 to take action on the death of Dr. Carlton C. Frederick.

*Section of Medicine.*—Tuesday evening, May 9. Program: "Robert Koch," DeLancey Rochester; Mycotic Disease—presentation of an unusual case of oidiomycosis, Henry C. Buswell. Pathologic findings of this case were presented by Charles A. Bentz.

*Section of Pathology.*—Tuesday evening, May 16. Program: Lumbar puncture, DeWitt H. Sherman; Report of 100 cases of Wassermann reaction, A. A. Thibaudeau.

*Section of Obstetrics and Gynecology.*—Tuesday evening, May 23. Program: Some points regarding pelvic operations, S. Y. Howell.

